# IL-6 triggers lysosomal degradation of LDL-R and enhances LDL-C uptake in vascular endothelial cells via macropinocytosis

**DOI:** 10.1186/s10020-026-01484-7

**Published:** 2026-04-25

**Authors:** Mulugeta M. Zegeye, Jishamol T. Veettil, Geena V. Paramel, Seta Kurt, Samira Salihovic, Liza U. Ljungberg, Ashok K. Kumawat, Allan Sirsjö

**Affiliations:** 1 Cardiovascular Research Centre (CVRC), School of Medical Sciences, Örebro University, Södra Grev Rosengatan 32, Örebro, 703 62 Sweden; 2 Department of Clinical Research Laboratory, Faculty of Medicine and Health, Örebro University, Örebro, Sweden; 3 Inflammatory Response and Infection Susceptibility Centre (iRiSC), School of Medical Sciences, Örebro University, Södra Grev Rosengatan 32, Örebro, 703 62 Sweden; 4 Present Address: Radcliffe Department of Medicine, Centre for Human Genetics, University of Oxford, Oxford, OX3 7BN UK

**Keywords:** Mylip/IDOL, Trans-signaling, Macropinocytosis, Atherosclerosis, Endothelial dysfunction

## Abstract

**Background:**

Endothelial dysfunction profoundly compromises the barrier function that precludes trans-endothelial entry of low-density lipoprotein cholesterol (LDL-C) into the vessel wall. LDL-C retention in the vessel wall is atherogenic and its flux involves several mechanisms including LDL-receptor (LDL-R) mediated transcytosis, a process that is facilitated by inflammatory stressors. In this study, we aimed to investigate the role of interleukin-6 (IL-6) in regulating LDL-R and LDL-C uptake by vascular endothelial cells.

**Method:**

We used commercially available Human umbilical vein endothelial cells (HUVECs) in this study. Flow cytometry, western blotting, qRT-PCR and ELISA were used to investigate expression of LDL-R and Mylip/IDOL. LDL-C uptake and free cholesterol levels in HUVECs was assessed using flowcytometry and mass-spectrometry respectively.

**Results:**

We show that HUVECs treated with a combination of IL-6 and soluble IL-6 receptor (sIL-6R) result in a significant reduction in surface expression of LDL-R, an effect that is reversed by soluble gp130Fc – an antagonist of IL-6 trans-singling. Using pharmacological inhibitors and gene silencing techniques, we demonstrate that IL-6 trans-signaling induced downregulation of LDL-R is attained through lysosomal degradation mediated by the E3 ubiquitin ligase Mylip. Conversely, HUVECs treated with IL-6 in combination with sIL-6R exhibit markedly increased uptake of native LDL-C which is also inhibited by sgp130Fc, the actin inhibitor Cytochalasin D and the macropinocytosis inhibitor EIPA. Although stimulation of HUVECs upregulated the expression of scavenger receptors CD36 and CXCL16, their contribution to native LDL-C uptake turned out to be negligible.

**Conclusion:**

Collectively, this study highlights the role of IL-6 in the regulation of LDL-R expression and cholesterol homeostasis in vascular ECs. IL-6 trans-signaling downregulates LDL-R yet increases LDL-C uptake via an LDL-R–independent, actin-dependent macropinocytosis pathway.

**Supplementary Information:**

The online version contains supplementary material available at 10.1186/s10020-026-01484-7.

## Background

Subendothelial retention of low-density lipoprotein cholesterol (LDL-C) is a pivotal and presumably rate-limiting step during atherosclerosis, a chronic inflammatory condition driving most of cardiovascular diseases (CVDs) (Gimbrone and García-Cardeña 2016; Rajendran et al., 2013). LDL-C retention in vascular intima is critically dependent on its passage across the endothelial layer, which under normal circumstances is a highly selective barrier (Gimbrone and García-Cardeña 2016; Rajendran et al., 2013). However, this barrier function is profoundly altered due to endothelial dysfunction and inflammation, increasing LDL-C entry into the intima and accelerating atherosclerotic plaque formation by setting off a cascade of inflammatory responses and foam cell formation, hallmarks of atherosclerosis (Kunjathoor et al., 2002; Yu et al., 2013).

Trans-endothelial LDL-C flux into arterial intima is mainly driven by fluid-phase transcytosis and a receptor-mediated transcytosis (Bolanle et al., 2025), where LDL-C binds to endothelial receptors such as scavenger receptor SR-BI (scavenger receptor class B type 1) and TGFβ family receptor ALK1 (activin receptor-like kinase 1) (Ghaffari et al., 2021; Armstrong et al., 2015; Kraehling et al., 2016). Although previously underappreciated, a recent study highlighted the significance of LDL-R mediated LDL-C transcytosis in endothelial cells activated by IL-1β, suggesting that the relative contribution of LDL-C transcytosis pathways is amenable to inflammatory stress (Jang et al., 2024).

The high affinity LDL-R is expressed on vascular endothelial cells, and its expression has been shown to be regulated by inflammatory stimuli including TNF-α and IL-1β (Jang et al., 2024; Zhang et al., 2014). In this study, we sought to investigate the role of IL-6, a pleotropic cytokine downstream of IL-1β, in regulating LDL-R and LDL-C uptake. While circulating IL-6 levels are generally low (typically < 10 pg/ml), IL-6 concentrations have been reported to increase by several orders of magnitude during inflammatory conditions, reaching ng/ml to even µg/ml levels (Rose-John 2012). Importantly, IL-6 has been identified as an independent predictor of CVDs, and it has been causally associated to atherosclerotic vascular diseases in several clinical studies (Zegeye et al., 2021; Kaptoge et al., 2014; Ridker et al., 2000; Niu et al., 2012). This is further recapitulated in experimental in vivo studies in which IL-6 has been shown to play indispensable role during atherogenesis (Hardin et al., 2011; Schuett et al., 2012). Furthermore, IL-6 has been directly linked to regulation of LDL-R gene expression and LDL-C uptake in hepatocytes and endothelial cells (Lubrano et al., 2015; Gierens et al., 2000), although how the distinct IL-6 signaling pathways regulate these processes remain poorly understood.

On molecular level, IL-6 mainly signals through two distinct mechanisms. The so-called IL-6 classic signaling, where IL-6 binds to a membrane-bound IL-6R that forms a heterodimer with gp130 signal transducer protein, has homeostatic and anti-inflammatory roles in vascular ECs and other cell types. In contrast, IL-6 trans-signaling, in which a complex of IL-6 and soluble IL-6R (sIL-6R) signals through gp130, is pro-inflammatory (Lindkvist et al., 2022; Ljungberg et al., 2020; Zegeye et al., 2020, 2018, 2023a). Human vascular ECs express low level of IL-6R and gp130 and can respond to both IL-6 classic- and trans-signaling pathways in distinctive manner (Zegeye et al., 2018; Montgomery et al., 2021; Ferreira et al., 2023).

Here, we report that IL-6 trans-signaling, but not classic-signaling, results in Mylip-dependent degradation of LDL-R in vascular endothelial cells while simultaneously upregulating LDL-C uptake through actin-mediated endocytic process tying IL-6 trans-signaling to enhanced fluid-phase LDL-C uptake.

## Methods and materials

### Cell culture

Human Umbilical Vein Endothelial cells (HUVECs, Life technologies, USA) were cultured in complete endothelial medium supplemented with VEGF Life factors (Lifeline cell Technologies, USA) and antibiotics (Penicillin and Streptomycin-PEST-Gibco, Life Technologies, USA) in 75cm^2^ flasks (Sarstedt, Germany). The cultures were incubated at 37 °C with 5% CO_2_ and cells were maintained until passage 10.

Prior to stimulation, HUVECs were seeded overnight at cell densities of 3 × 10^5^ cells/well in 6-well plates and 6 × 10^4^ cells/well in 24-well plates containing complete endothelial medium containing antibiotics. The next day, the media was replaced with antibiotic free media with or without the specific stimuli and incubated for the specified timepoints. At the end of incubations, the cells were either immediately used for analyses or stored at −80 °C until analyses. For experiments involving gene silencing, HUVECs were seeded at cell densities of 2 × 10^5^ cells/well in 6-well plates overnight in complete endothelial medium containing antibiotics. After an overnight incubation, the cells were rinsed with Opti-MEM (Gibco, Life Technologies, USA) followed by incubation with 700 μl Opti-MEM/well containing 2 μl lipofectamine (Invitrogen, USA) and a Silencer®Select siRNA against the target gene (0.5 nM siRNA, Invitrogen, USA). Cells incubated with non-target Silencer®Select siRNA (0.5 nM, Invitrogen, USA) were included as controls. After 4 h of incubation, 1.3 ml of complete endothelial medium was added into each well and incubation continued, after which culture media and cells were collected and kept at −80 °C until further analysis.

### Flowcytometry analyses

HUVECs were detached and washed twice with PBS containing 1 mM EDTA and 2% FBS and then stained with anti-LDL-R-APC antibody (clone: 301, Abcam, UK), anti-CD36-FITC antibody (clone: 5–271 from BioLegend supplied by Nordic Biosite, Sweden) or anti-CXCL16-PE antibody (clone: 22–19-12 from BioLegend supplied by Nordic Biosite, Sweden) for 25 min at 4 °C in the dark. Fluorescence minus one (FMO) control were included as negative control for staining. To remove dead cells from analysis, 7AAD staining was employed. Stained cells were acquired using Gallios™ flow cytometer (Beckman Coulter, Brea, CA, USA) and analyzed using Kaluza flow cytometry analysis software version 1.3 (Beckman Coulter, Brea, CA, USA).

### Western blotting

Using ice-cold RIPA lysis buffer (Millipore, USA), HUVECs were lysed, and the protein content of the lysate was quantified using Micro BCA™ Assay kit (Thermo Fisher Scientific, USA) according to the manufacturer’s instructions. The cell lysate was mixed with an SDS sample buffer followed by denaturation for 5 min at 95 °C. Electrophoretic separation was achieved by loading 10–20 μg of protein/well into NuPAGE® (4–12%) Novex Bis–Tris gels with ice-cold MOPS running buffer (Invitrogen, USA) along with MagicMark™ XP Western Protein Standard (Invitrogen, USA), which was used to determine molecular masses of the proteins. A constant voltage of 140 V was applied to achieve separation, after which the proteins were transferred onto Immobilon-FL PVDF membranes (Millipore, USA) by applying a constant current of 125 mA/membrane. Membranes were incubated with primary antibodies and respective HRP- conjugated secondary antibodies. The primary antibodies used were anti-LDL-R (Abcam, UK, ab52818; 1:1000 dilution), anti-Mylip antibody (LS-C226906 from LSBio, supplied by Nordic Biosite, Sweden; 1:1000 dilution) and Anti-β Tubulin antibody (Abcam, UK, ab231082; 1:1000 dilution). This was followed by incubation with horseradish peroxidase (HRP)-conjugated goat anti-rabbit IgGs (Cell Signaling Technology, USA, #7074; 1:2000) or horse anti-mouse IgGs (Cell Signaling Technology, USA, #7076; 1:2000). Visualization of the bands was achieved by using Immobilon™ Western Chemiluminescent HRP Substrate solution (Millipore, USA), and chemiluminescence was recorded by a Li-Cor Odyssey Fc imager and analyzed with Image Studio Software (Li-Cor Biotechnology UK Ltd., United Kingdom).

### Total RNA extraction, cDNA synthesis and qPCR

Extraction of total RNA was achieved using E.Z.N.A® Total RNA kit (Omega Bio-Tek, Norcross, GA, USA) according to the manufacturer’s instructions. The quantity and quality of RNA extract was determined using NanoDrop™ 2000 (Thermo Fisher Scientific, USA) spectrophotometer. Then, cDNA was synthesized from the extracted RNA using high-capacity cDNA reverse transcription kit (Thermo Fisher Scientific, USA) following the manufacturer’s instructions. Briefly, 1 μg RNA extract was mixed with a master mix containing random primers, reverse transcriptase enzyme, dNTPs and buffer. The final volume was adjusted to 20 µl using nuclease free water and the preparation was allowed to run in a thermal cycler for: 10 min at 25 °C, 120 min at 37 °C, 5 min at 85 °C and kept at 4 °C before storage at − 20 °C. For gene expression studies, we used TaqMan qPCR primers/probes (Applied Biosystems, Life technologies, USA), and QuantStudio 7 Flex Realtime PCR system (Applied Biosystems,USA). GAPDH was used as housekeeping gene for normalization.

### Lipidomics/mass-spectrometry

Cell pellets contained in Eppendorf tubes were spiked (10 ul) with stable isotope labeled (SIL) standard Cholesterol-d7 (Avanti Lipids, Alabaster, AL). Extraction was performed using protein precipitation with 490ul of pre-cooled (−20 °C) isopropanol. Samples were vortexed mixed for 1 min and placed at −20 °C for 10 min. Samples were vortex mixed again for 1 min and placed at 4 °C for 2 h to ensure complete protein precipitation. The extracted samples were centrifuged at a maximum of 10,300 g for 10 min at 4 °C before transferring the supernatant to total recovery glass vials. Ultra-high pressure liquid chromatography-tandem mass spectrometry measurements were performed on an Acquity UHPLC coupled to a Xevo TQ-XS triple quadrupole mass spectrometer (MS/MS) (Waters Corporation, Milford, MA, USA). Two µL of the sample extract was injected onto a 1.7 µm, 2.1 mm × 150 mm Acquity BEH AMIDE column, in combination with a 1.7 µm, 2.1 mm × 5 mm BEH Amide VAN GUARD column. Mobile phase was composed of A = 10 mM ammonium acetate in Acetonitrile:H2O (95:5) and B = 10 mM ammonium acetate in Acetonitrile:H2O (50:50). The linear gradient elution from 0.1% to 20% B was applied for 2 min, from 20 to 80% B for 3 min, followed by 3 min of re-equilibration to the initial chromatographic conditions. The flow rate was 0. 600 ml/min, column temperature 45 °C. The analysis was performed using positive mode unispray ionization. The source temperature was 150 °C and the desolvation temperature was 500 °C. The cone gas flow was 150 L/h and the desolvation gas flow was 1000 L/h. The multiple reaction monitoring (MRM) acquisition mode was selected for the absolute quantification of the targeted analytes with an individual span time of 0.1 s, given in their individual MRM channels. Nitrogen was used as a nebulizer and argon was used as collision gas. Relative quantification, normalized to internal standard, is reported.

### Sandwich enzyme linked immuno-sorbent assay (ELISA)

DuoSet® ELISA kit (R&D Systems, Minneapolis, USA) was used to quantify soluble LDL-R (sLDL-R) from endothelial cell culture supernatants, following manufacturers instruction. Briefly, a 96-well plate was coated with a PBS-dissolved capture antibody overnight. All washings in between each step were performed using PBS containing 0.05% Tween (PBS-T) and all incubation were made at room temperature. After capture antibody coating and washing, PBS-T diluted standards and samples are added to the wells and incubated for 2 h. Following that, the wells were incubated with a biotinylated-conjugated secondary antibody for 2 h, an HRP solution for 20 min and substrate solution for 20 min after which the reaction is stopped by adding 2 N H_2_SO_4_. Finally, absorbance was read at 450 nm using Cytation 3 plate reader (BioTek, Winooski, USA).

### LDL-C uptake assay

LDL-C uptake assay was performed using pHrodo™ Red-LDL conjugates (Thermo Fisher Scientific, USA) following HUVECs stimulation according to respective experiments. Briefly, HUVECs were incubated with 5 µg/ml pHrodo™ Red-LDL conjugate dissolved in Fluorobrite™ DMEM medium (Invitrogen, USA) for 3 h in the presence or absence of inhibitors including sgp130Fc, sLDL-R, Cytochalasin D and EIPA (5-(N-Ethyl-N-isopropyl)amiloride). The fluorescence signal generated by the pHrodo™ Red labeled LDL upon internalization into intracellular acidic compartments was measured to determine LDL-C uptake. Assessment of LDL-C uptake was done using two approaches, microscopy and flowcytometry. For microscopy, the cells were washed with PBS and then fluorescence and bright field images were taken using Cytation 3 plate reader (BioTek, Winooski, USA). For flowcytometry analyses, cells were washed with PBS containing 1 mM EDTA and 2% FBS (for flowcytometry) and events were collected using Gallios™ flow cytometer (Beckman Coulter, Brea, CA, USA). The data was analyzed using Kaluza flow cytometry analysis software version 1.3 (Beckman Coulter, Brea, CA, USA).

### Dextran uptake assay

HUVECs were incubated with 1 mg/ml Dextran-Texas Red (70 kDa) conjugate dissolved in Fluorobrite™ DMEM medium (both from Invitrogen, USA) for 1 h in the presence or absence of EIPA. HUVECs were then washed with PBS containing 1 mM EDTA and 2% FBS and events were collected using Gallios™ Flow Cytometer (Beckman Coulter Life Sciences, UK). The data was analyzed using Kaluza flow cytometry analysis software version 1.3 (Beckman Coulter, UK).

### Statistical analysis

Data are presented as mean ± standard error of mean (SEM) of at least 3 sets of independent experiments, and analyses were performed using GraphPad Prism® statistical software version 10.0 (GraphPad Software, Inc., USA). Statistical tests such as one-way ANOVA with repeated measures followed by Bonferroni post-hoc test, paired t-test, and One sample t-test were used to compare groups. A *p*-value less than 0.05 was considered as statistically significant.

## Results

### IL-6 trans-signaling, but not classic-signaling, reduces expression of LDL-R in human vascular endothelial cells

**Fig. 1:**
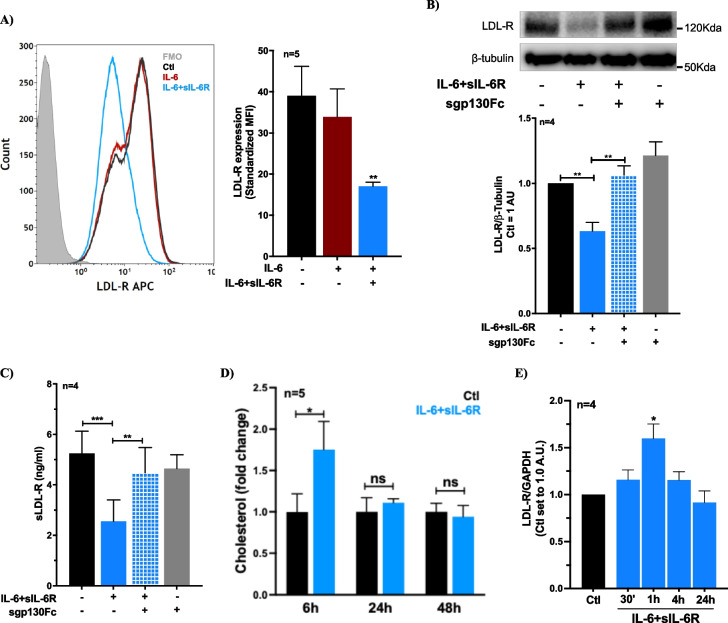
IL-6 trans-signaling downregulates expression of LDL-R in human vascular ECs. **A** Histogram and a bar graph showing surface expression of LDL-R using flowcytometry in ECs after 48 h of treatment with IL-6 (100 ng/ml) with or without sIL-6R (100 ng/ml). **B** Representative immunoblots and a bar graph showing LDL-R expression in ECs treated with a combination of IL-6 and sIL-6R (100 ng/ml each) with or without sgp130Fc (1 µg/ml). **C** A bar graph shows that IL-6 + sIL-6R (100 ng/ml each) induced reduction (48 h) in release of sLDL-R from ECs is inhibited by addition of sgp130Fc (1 µg/ml). **D** A bar graph showing relative abundance of cholesterol (LC–MS/MS) in unstimulated ECs versus ECs treated with a combination of IL-6 and sIL-6R (100 ng/ml each). Cholesterol levels were normalized to total protein amount determined by BCA assay. A paired t-test was employed. **E** A bar graph showing the LDL-R gene expression in ECs treated with IL-6 + sIL-6R (100 ng/ml each) for 30 min to 24 h. A one-way ANOVA followed by Bonferroni post-hoc test was employed. *p < 0.05, **p < 0.01, ***p < 0.001

To investigate whether IL-6 regulates expression of LDL-R in ECs, we stimulated cultured HUVECs with recombinant human IL-6 and soluble IL-6R (sIL-6R). To simulate IL-6 classic-signaling activation, HUVECs were treated with IL-6 alone while IL-6 trans-signaling activation was induced by adding a combination of IL-6 and sIL-6R (IL-6 + sIL-6R). The concentration for both recombinant proteins was 100 ng/ml, which was chosen based on our previous dose–response experiments (Zegeye et al., 2018). Flowcytometry analyses of HUVECs after 48 h of stimulation showed that surface expression of LDL-R was significantly reduced in ECs treated with IL-6 + sIL-6R compared to untreated controls (Fig. 1A). However, LDL-R surface expression in ECs treated with IL-6 alone was not affected. Moreover, using immunoblotting and flowcytometry, we showed that the reduction in LDL-R expression caused by IL-6 + sIL-6R was inhibited by addition of sgp130Fc (Fig. 1B and supplementary Fig. 1), which selectively inhibits activation of IL-6 trans-signaling. These findings show that IL-6 trans-, but not classic-signaling, significantly reduces expression of LDL-R in human vascular ECs.

In our previous study, we reported that endothelial LDL-R can be cleaved and released as a soluble form by the peptidases ADAM17 and MMP14 in response to the pro-inflammatory cytokine TNF-α (Zegeye et al., 2023b). Here we assessed whether activation of IL-6 trans-signaling in ECs could also induce shedding thereby suppressing the surface level of LDL-R. We analyzed cell culture supernatants of ECs treated with or without IL-6 and IL-6 + sIL-6R using ELISA. As shown in Fig. 1C, the concentration of sLDL-R was significantly reduced in ECs treated with IL-6 + sIL-6R compared to untreated controls. Supernatant from ECs treated with IL-6 alone, however, showed similar level of sLDL-R to that of untreated controls (supplementary Fig. 2). Furthermore, the reduction in sLDL-R in response to IL-6 + sIL-6R treatment was blocked by addition of sgp130Fc confirming that the effect is IL-6 trans-signaling dependent (Fig. 1C). Because LDL-R is centrally regulated by availability of intracellular cholesterol (Goldstein et al., 2006), we assessed whether IL-6 trans-signaling alters level of free cholesterol in ECs. Indeed, mass-spectrometry analyses using ECs treated with IL-6 + sIL-6R showed a transient increase in cholesterol levels (6 h) compared to untreated controls (Fig. 1D). However, qPCR analysis revealed that LDL-R reduction due to IL-6 trans-singling was not due to transcriptional downregulation of the LDL-R gene expression (Fig. 1E). Collectively, these findings indicate that IL-6 trans-signaling mediated reduction of LDL-R expression is less likely due to accumulation of unesterified cholesterol and not due to shedding or transcriptional downregulation.

### IL-6 trans-signaling causes Mylip-dependent lysosomal degradation of LDL-R in human vascular endothelial cells

**Fig. 2:**
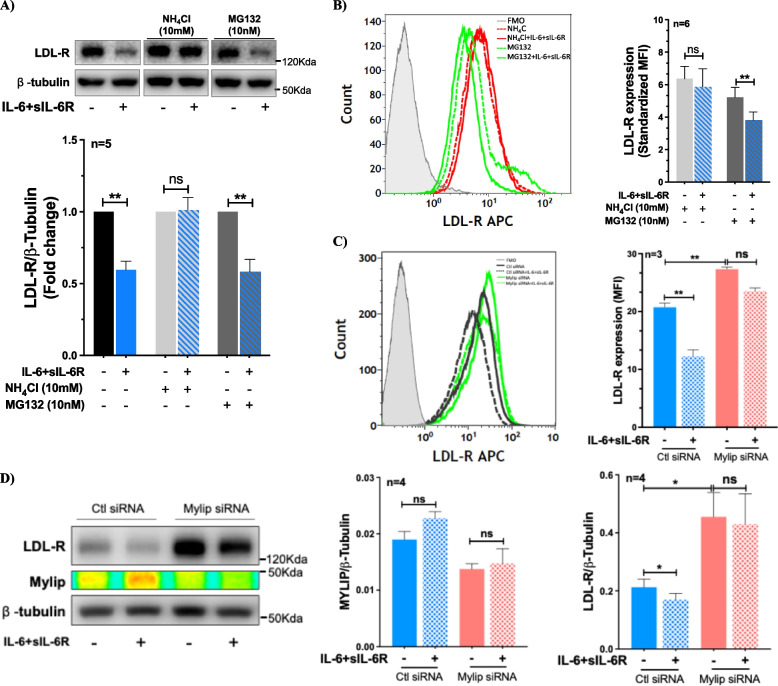
IL-6 trans-signaling downregulates endothelial LDL-R through Mylip-dependent lysosomal degradation. ECs were treated with a combination of IL-6 and sIL-6R (100 ng/ml each) with or without NH_4_Cl (10 mM) and MG132 (10 nM) for 48 h. **A** Representative immunoblots and a bar graph showing LDL-R expression in ECs. A one-sample t-test was used. **B** Histogram and a bar graph showing surface expression of LDL-R using flowcytometry in ECs. **C** Histogram and a bar graph showing surface expression of LDL-R using flowcytometry in ECs following Mylip gene knockdown and stimulation with IL-6 + sIL-6R (100 ng/ml each). **D** Representative immunoblots and bar graph showing LDL-R and Mylip expression in ECs following Mylip gene knockdown and stimulation with IL-6 + sIL-6R (100 ng/ml each). A one-way ANOVA followed by Bonferroni post-hoc test was employed. *p < 0.05, **p < 0.01

Next, we investigated whether IL-6 trans-signaling in ECs employs post-transcriptional pathways to downregulate LDL-R expression using pharmacological inhibitors of lysosomal (NH_4_Cl) and proteasomal (MG 132) degradation pathways in ECs treated with IL-6 + sIL-6R. Co-stimulation of ECs with IL-6 + sIL-6R and MG132 resulted in a significant reduction in LDL-R level. However, ECs treated with IL-6 + sIL-6R in the presence of NH_4_Cl showed no significant change in the level of LDL-R suggesting that IL-6 trans-signaling downregulated LDL-R level by enhancing its lysosomal degradation (Fig. 2A and B).

Post-translational regulation of LDL-R is known to be driven by PCSK9 (Proprotein convertase subtilisin/kexin type 9), which binds to LDL-R and directs it to lysosomal degradation, and Mylip, an LXR-induced E3 ubiquitin ligase that ubiquitinates LDL-R targeting it for endosomal sorting and lysosomal degradation (Zhang et al., 2007; Zelcer et al., 2009). Here, we found that PCSK9 expression in ECs was below detection threshold of qPCR; and treatment of ECs with IL-6 + sIL-6R (30 min-24 h) showed no apparent upregulation of PCSK9 gene expression. In addition, ECs treated with IL-6 + sIL-6R (30 min-24 h) showed no apparent change in gene expression of Mylip (supplementary Fig. 3A). However, we found a trend towards an increase in the level of Mylip protein in ECs following stimulation with IL-6 + sIL-6R (mean difference = 0.004, *p*-value = 0.216) although it did not reach statistical significance (Fig. 2D). To clarify whether Mylip is responsible for IL-6 trans-signaling induced LDL-R downregulation in ECs, we performed gene silencing using siRNA that targeted the Mylip gene resulting in ~ 70% knockdown efficiency (supplementary Fig. 3B). As expected, the silencing of Mylip caused an upregulation of LDL-R expression, consistent with its reported involvement in homeostatic LDL-R regulation (Fig. 2C-D and supplementary Fig. 3C). Furthermore, the knockdown of Mylip reversed the IL-6 + sIL-6R induced reduction in LDL-R expression (Fig. 2C and D). Collectively, these findings demonstrate that IL-6 trans-signaling induced LDL-R downregulation is driven through Mylip-dependent lysosomal degradation.

### IL-6 trans-signaling enhances LDL-C uptake by ECs despite low levels of LDL-R

**Fig. 3:**
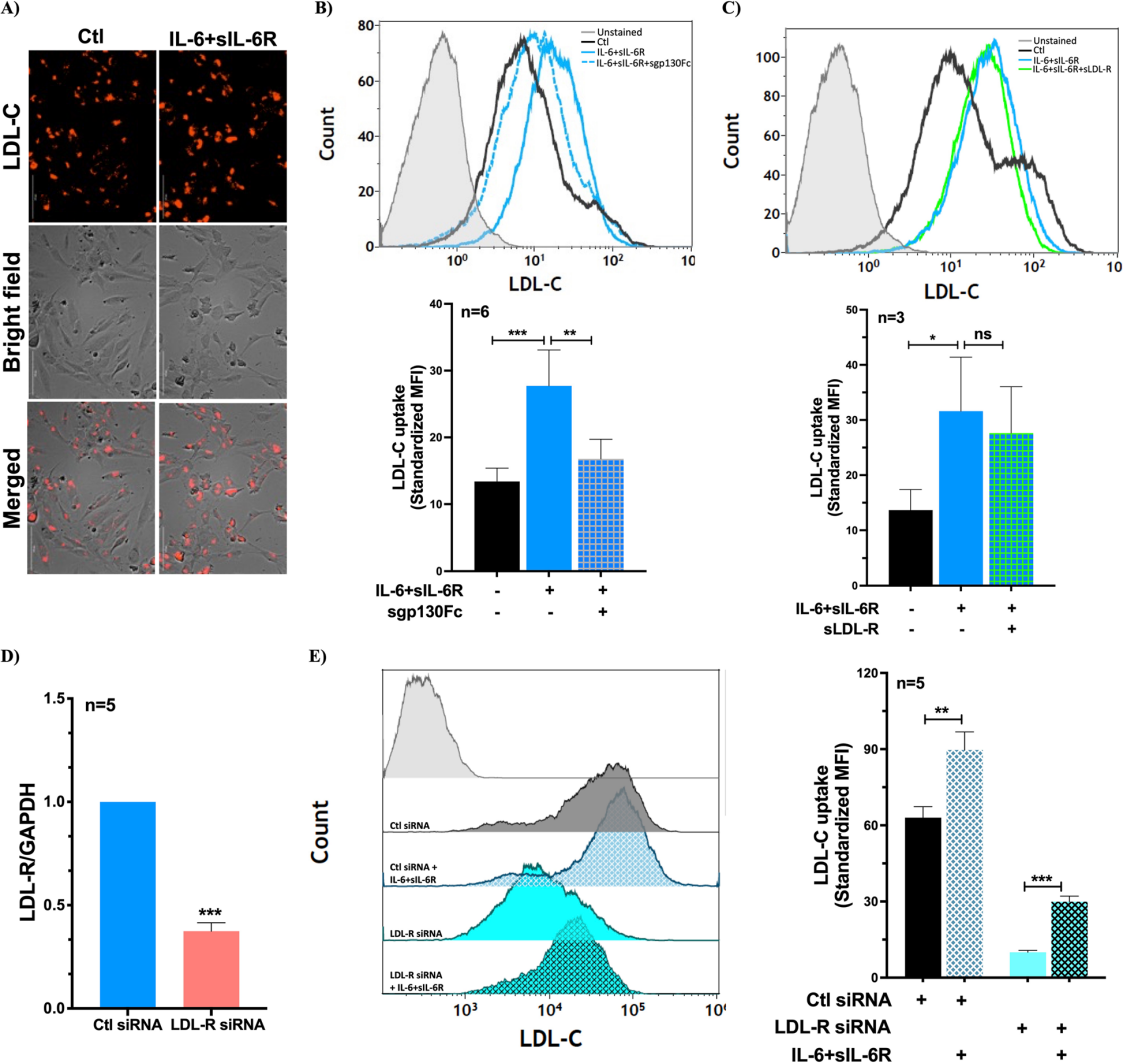
IL-6 trans-signaling activation in ECs enhances LDL-C uptake in an LDL-R independent manner. HUVECs were treated with IL-6 + sIL-6R (100 ng/ml each) for 48 h following which the cells were incubated with pHrodo Red conjugated LDL-C for 3 h. **A** Fluorescence signal of pHrodo Red conjugated LDL-C and bright field images of HUVECs. Representative histograms and bar graphs show LDL-C uptake by HUVECs treated with IL-6 + sIL-6R (100 ng/ml each) in the presence or absence of **B**) sgp130Fc (1 µg/ml) and **C**) sLDL-R (100 µM). **D** Bar graph showing knockdown efficiency of LDL-R in ECs exposed to targeting siRNAs as compared to ECs exposed to scramble siRNA. **E** Representative histogram and a bar graph show LDL-C uptake by HUVECs following LDL-R gene knockdown and stimulation with IL-6 + sIL-6R (100 ng/ml each). A one-way ANOVA followed by Bonferroni post-hoc test was employed. *p < 0.05, **p < 0.01, ***p < 0.001

To investigate the functional consequence of IL-6 trans-signaling induced reduction of LDL-R on surface of ECs, we assessed LDL-C uptake by ECs after 48 h of treatment with IL-6 alone or in combination with sIL-6R. We found that ECs treated with IL-6 + sIL-6R had significantly elevated level of LDL-C uptake compared to untreated controls (Fig. 3A-C), meanwhile, IL-6 treated ECs showed similar level of LDL-C uptake as untreated controls (supplementary Fig. 4). Moreover, the increased LDL-C uptake by ECs exposed to IL-6 + sIL-6R was abolished using sgp130Fc (Fig. 3B). These findings suggest that the increased LDL-C uptake in response to IL-6 is specific to activation of trans-signaling. Furthermore, we assessed the contribution of residual LDL-R activity to the enhanced LDL-C uptake induced by IL-6 + sIL-6R using two approaches: inhibition of LDL-R-mediated uptake using sLDL-R, as shown previously (Zegeye et al., 2023b), and silencing LDL-R gene expression. We found that addition of sLDL-R in combination with IL-6 + sIL-6R did not alter the increase in LDL-C uptake by ECs (Fig. 3C). Similarly, silencing LDL-R gene expression (~ 65% knockdown efficiency, Fig. 3D) did not affect IL-6 + sIL-6R induced increase in LDL-C uptake, although it significantly reduced basal LDL-C uptake (Fig. 3E). Taken together, these findings indicate that IL-6 trans-signaling-induced increase in LDL-C uptake by ECs is independent of LDL-R activity.

**Fig. 4:**
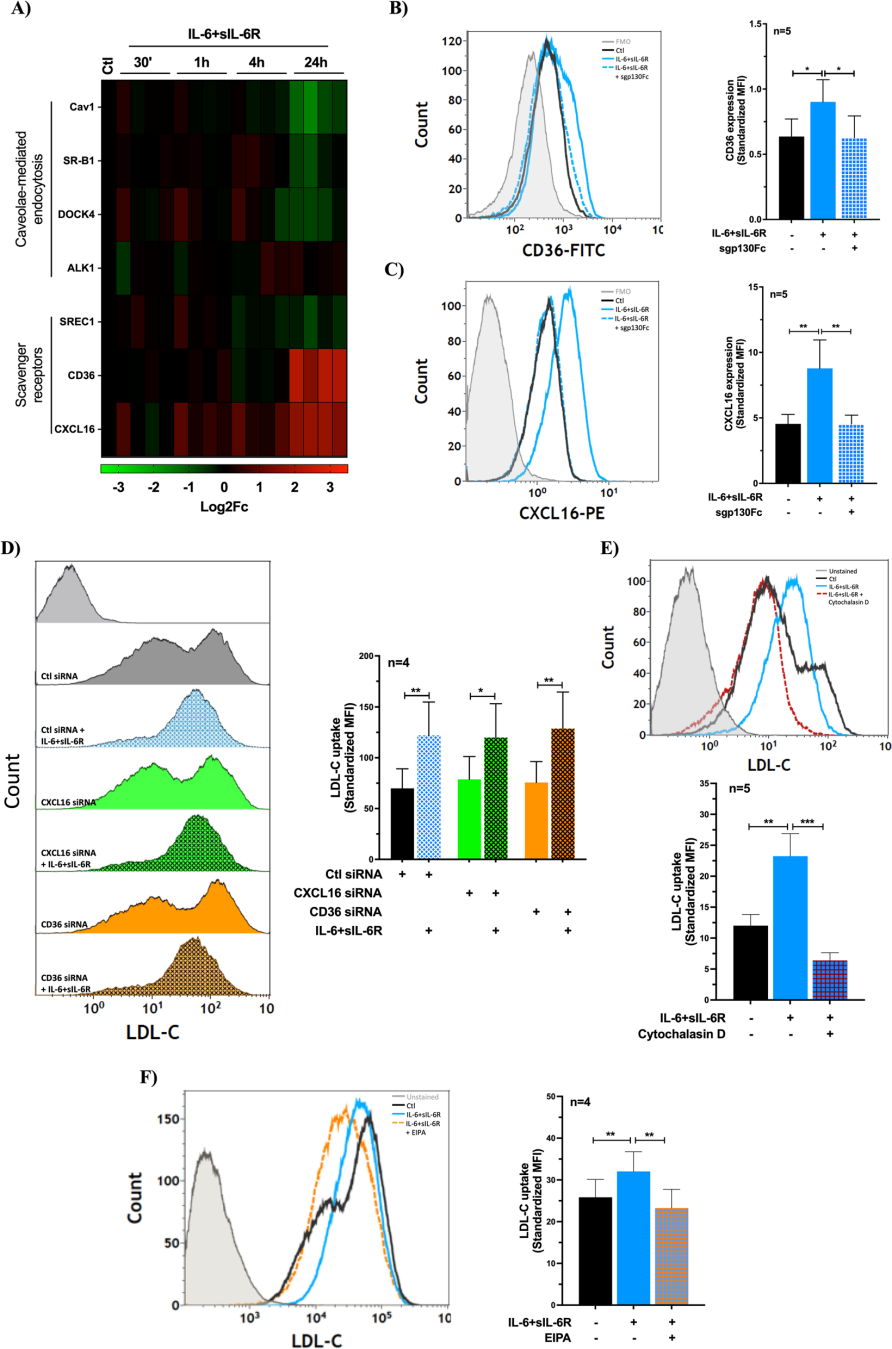
The uptake of LDL-C in ECs induced by IL-6 trans-signaling occurs via actin-dependent fluid-phase macropinocytosis pathway, with limited involvement of scavenger receptors or caveolae-mediated endocytosis. **A** Heatmap showing the endothelial gene expression of scavenger receptors and genes involved in caveolae-mediated endocytosis in response to treatment with IL-6 + sIL-6R (100 ng/ml each). Representative histograms and bar graphs show surface expression of **B**) CD36 and **C**) CXCL16 in HUVECs treated with IL-6 + sIL-6R and sgp130Fc (1 µg/ml) for 48 h. Representative histograms and bar graphs show IL-6 trans-signaling induced LDL-C uptake in **D**) ECs lacking either CXCL16 or CD36 expression, in **E**) ECs treated with Cytochalasin D (1 µM), and in **F**) ECs treated with EIPA (25 µM). A one-way ANOVA followed by Bonferroni post-hoc test was employed. *p < 0.05, **p < 0.01, ***p < 0.001

Beside LDL-R mediated pathway, LDL-C can be taken up by ECs through caveolae mediated endocytosis, scavenger receptors and pinocytosis. Therefore, we sought to investigate whether IL-6 trans-signaling causes an upregulation of genes involved in any of those pathways that may account for the increased LDL-C uptake. As shown in Fig. 4A and Supplementary Fig. 5, stimulation of ECs with IL-6 + sIL-6R resulted in significant upregulation of the scavenger receptors CD36 and CXCL16. Because both CD36 and CXCL16 have soluble forms, we assessed whether these receptors are expressed on the surface of ECs using flowcytometry. We showed that both CD36 and CXCL16 are expressed on the surface of ECs and the upregulation induced by IL-6 trans-signaling could be abolished by treatment with sgp130Fc (Fig. 4B and C). However, no apparent changes were seen on the expression of genes involved in caveolae-mediated LDL-C endocytosis. CD36 has been shown to bind native LDL-C although CXCL16 is only capable of binding to modified LDL-C (Calvo et al., 1998; Shimaoka et al., 2000). Therefore, we set out to determine the role of CD36 and CXCL16 in IL-6 trans-signaling induced uptake of LDL-C in ECs. Using siRNAs, expression of CD36 and CXCL16 was silenced (~ 80–90% knockdown efficiency, Supplementary Fig. 6) and the IL-6 trans-signaling induced LDL-C uptake was analyzed. It turned out that silencing of neither CD36 nor CXCL16 could inhibit IL-6 trans-signaling induced uptake of LDL-C by ECs (Fig. 4D). However, when ECs were treated with IL-6 + sIL-6R in the presence of actin inhibitor cytochalasin D, the IL-6 trans-signaling induced increase in LDL-C uptake was inhibited (Fig. 4E). Furthermore, using EIPA, a specific macropinocytosis inhibitor, we found that the IL-6 trans-signaling induced increase in LDL-C as well as Texas-red-labeled dextran (70kDa) uptake was inhibited (Fig. 4F and Supplementary Fig. 7). Overall, our findings indicate that IL-6 trans-signaling increases endothelial LDL-C uptake through actin-dependent macropinocytosis with limited involvement of scavenger receptors or caveolae-mediated endocytosis, and despite downregulated LDL-R expression.

## Discussion

This study highlights the role of IL-6 in the regulation of LDL-R expression and cholesterol homeostasis in vascular ECs, linking activation of the pro-inflammatory IL-6 trans-signaling pathway to suppression of LDL-R level alongside enhanced accumulation of unmodified LDL-C in ECs through actin-dependent, receptor-independent macropinocytosis pathway.

We showed that activation of IL-6 trans-signaling, but not classic-signaling, causes significant reduction on the surface expression of endothelial LDL-R through Mylip-dependent lysosomal degradation. Mylip, also known as IDOL (inducible degrader of LDL-R) is a post-translational regulator of LDL-R which, unlike PCSK9, is abundantly expressed in ECs (Schaum et al., 2018; Karlsson et al., 2021). Mylip is a direct transcriptional target of the liver X receptors (LXR), a transcription factor that is classically regulated by sterols (Zhang et al., 2012), but also by pro-inflammatory cytokines such as IL-1β and TNF-α (Kim et al., 2007). In our case, IL-6 trans-signaling in ECs did not alter the gene expression of Mylip; it rather is associated with a non-significant trend toward increased protein abundance, which coincided with the reduction in LDL-R. Mylip level is known to be increased by post-transcriptional mechanisms such as SUMOylation that inhibits autoubiquitination and degradation (Wang et al., 2021). IL-6 has been shown to modulate SUMOylation pathways thereby regulating downstream processes (Ohbayashi et al., 2008). Although not examined in our study, such mechanisms could explain the small increase in Mylip protein observed upon IL-6 trans-signaling and merit further study. Our findings are particularly relevant because the major source of Mylip in tissues such as the liver appears to be ECs suggesting for a potential paracrine effect (Schaum et al., 2018; Karlsson et al., 2021). Furthermore, it has been reported that circulating Mylip is positively correlated with high plasma LDL-C (Girona et al., 2018). However, whether circulating Mylip directly influences hepatic LDL-R thereby decreasing LDL-C clearance remains to be investigated.

Under normal physiological condition, the level of intracellular cholesterol controls the activity of SREBP2 (sterol regulatory element-binding protein 2), a principal transcription factor that regulates expression of LDL-R (Brown and Goldstein 1997). SREBP2 is activated when intracellular cholesterol is low while it remains inactive when there is abundant free cholesterol. In our study, it turns out that the level of free cholesterol in ECs was transiently increased following IL-6 trans-signaling although the gene expression of LDL-R was not affected during the that time frame. However, at a later timepoint (48 h), the level of LDL-R was significantly downregulated in response to IL-6 trans-signaling, while the intracellular free cholesterol level was unaltered suggesting that the IL-6 trans-signaling induced LDL-R downregulation is not due to cholesterol mediated feedback regulation. These observations extend current understanding demonstrating that pro-inflammatory stressors can override sterol-sensing dependent regulation of LDL-R ultimately disrupting cellular feedback mechanisms (Jang et al., 2024; Zhang et al., 2014; Fowler et al., 2022). The contribution of IL-6 trans-signaling mediated receptor shedding was ruled out as the soluble LDL-R was also markedly reduced, although IL-6 is known to increase expression of shedding enzymes such as MMP14 (Cathcart et al., 2016).

Our findings reveal that IL-6 trans-, but not classic-signaling, increases endothelial unmodified LDL-C uptake in addition to its previously reported pro-inflammatory and pro-atherogenic effects (Zegeye et al., 2020, 2018, 2023a). It has been extensively studied that excessive accumulation of LDL-C in vascular ECs leads to disrupted barrier integrity, reduced nitric oxide production, increased oxidative stress and inflammatory responses that collectively promote endothelial dysfunction, increased entry of LDL-C into vascular intima and atherosclerosis (Mundi et al., 2018; Zanoni et al., 2018). Besides, in vitro and in vivo studies have shown that, ECs can generate cholesterol crystals under hyperlipidemic conditions and deposit it into the subendothelial layer (Baumer et al., 2017). Cholesterol crystals are abundant in plaques directly contributing to the pathogenesis of atherosclerosis in several mechanisms including induction of inflammatory responses in ECs as well as smooth muscle cells (Thazhathveettil e al., 2025; Thazhathveettil et al., 2024; Baumer et al., 2025).

Mechanistically, we showed that LDL-C uptake induced by IL-6 trans-signaling is independent of LDL-R activity as the uptake remained significantly elevated despite downregulation of LDL-R expression using siRNA-guided gene silencing as well as inhibition of the receptor-mediated uptake using sLDL-R. However, we demonstrated that the uptake is suppressed by the actin inhibitor Cytochalasin D and the macropinocytosis inhibitor EIPA, suggesting that the process is mainly driven by LDL-R-independent, fluid-phase macropinocytosis (Michael et al., 2013). LDL-C uptake via fluid-phase endocytic routes takes place in endothelial cells in an unsaturable manner as indicated in earlier electron microscopy findings and subsequent studies (Vasile et al., 1983; Zhang et al., 2018). Endocytosis of LDL-C particles represents intermediate event in their transcytosis across endothelial cells, which recent evidence suggests is the dominant pathway for LDL-C transport across arterial endothelium (Bolanle et al., 2025; Zhang et al., 2014). Although it was not tested directly in the current study, it is possible that the internalized LDL-C may undergo transcellular trafficking to be released to the opposite side of endothelial cells. Hence, our findings imply that non-receptor mediated endocytic pathways can increase entry of native LDL-C into vascular intima particularity when excess LDL-C is combined with an inflammatory stress. Moreover, if enhanced endothelial LDL-C uptake in response to IL-6 tans-signaling is confirmed in vivo, it may provide a potential mechanistic link explaining how unmodified LDL-C accumulates with in the intima, where it can subsequently be internalized by macrophages through non-saturable pinocytosis and contribute to foam cell formation as reported previously (Barthwal et al., 2013). Overall, inflammatory stress-induced LDL-C uptake in vascular ECs may represent a potential area for future investigation, complementing lipid lowering therapies, pending validation in more physiologically relevant cell models such as human aortic ECs as well as in vivo models. Notably, a recent study employing a genetic and pharmacologic inhibition of fluid-phase macropinocytosis in macrophages demonstrated a markedly reduced atherosclerotic lesion development in murine models of atherosclerosis (Lin et al., 2022).

Inflammatory stressors are known to regulate the scavenger receptors CD36 and CXCL16 in macrophages and vascular smooth muscle cells causing oxidized LDL-C uptake and foam cell formation (Wågsäter et al., 2004; Wuttge et al., 2004; Wuttge et al., 2001). Intriguingly, IL-6 trans-signaling increased expression of CD36 and CXCL-16 although their contribution towards the IL-6 trans-signaling-induced uptake of unmodified LDL-C was negligible. Nevertheless, these receptors, particularly CXCL16 that primarily recognize modified LDL-C, may potentially contribute towards uptake and accumulation of modified LDL-C in vascular ECs thereby promoting atherosclerosis (Shimaoka et al., 2000). Furthermore, endothelial CXCL-16 is known to promote recruitment of CXCR-6 expressing immune cells to vascular intima contributing to plaque progression (Collado et al., 2018; Liu et al., 2025). Overall, these findings show the multi-faceted detrimental effects of IL-6 trans-signaling on vascular ECs.

## Supplementary Information

Supplementary Material 1.

## Data Availability

The data used and/or analyzed in this study are contained within the manuscript.

## Abbreviations

ADAM17: A disintegrin and metalloprotease 17
ALK1: Activin receptor-like kinase 1
CVDs: Cardiovascular diseases
ECs: Endothelial cells
EIPA: 5-(N-Ethyl-N-isopropyl) amiloride
FMO: Fluorescent-minus-one
gp130: Glycoprotein 130
HUVECs: Human umbilical vein endothelial cells
IDOL: Inducible degrader of LDL-receptor
IL-6: Interluekin-6
IL-6R: Interluekin-6 receptor
LDL-C: Low-density-lipoprotein cholesterol
LDL-R: Low-density-lipoprotein receptor
LXR: Liver-x-receptor
MMP14: Matrix metalloprotease 14
PCSK9: Proprotein convertase subtilisin/kexin type 9
sgp130Fc: Soluble glycoprotein 130-Fragment crystallizable
sIL-6R: Soluble Interluekin-6 receptor
SR-BI: Scavenger receptor class B type I
SREBP2: Sterol regulatory element-binding protein 2
