# Supplementary material for: IL-6 triggers lysosomal degradation of LDL-R and enhances LDL-C uptake in vascular endothelial cells via macropinocytosis
Source: Mol Med. 2026 Apr 25;32:65. doi: 10.1186/s10020-026-01484-7 (PMC13109890; doi:10.1186/s10020-026-01484-7)
Supplement: Supplementary file 1 — Supplementary Material 1. [file 10020_2026_1484_MOESM1_ESM.docx]

**Supplementary data**

| **** | **** |
| --- | --- |
| **Supplementary figure 1.** Reduction in LDL-R expression following IL-6 trans-signaling activation in ECs is blunted by co-stimulation with sgp130FC. Histogram and a bar graph showing surface expression of LDL-R using flowcytometry in ECs after 48h of treatment with IL-6+sIL-6R (100ng/ml each) with or without sgp130Fc (1µg/ml). *p<0.05, ***p<0.001. | |

| **** |
| --- |
| **Supplementary figure 2.** Concentration of soluble LDL-R (sLDL-R) in endothelial cell culture media is altered by treatment with IL-6+sIL-6R, but not with IL-6 alone. A concentration of 100ng/ml is used for both IL-6 and sIL-6R for 48h of incubation. **p<0.01. |

| **A)**  **** | **B) ** | **C)**  **** |
| --- | --- | --- |
| **Supplementary figure 3.** A) Bar graph showing gene expression of Mylip in ECs treated with IL-6+sIL-6R for 30min upto 24h. B) Bar graph showing knockdown efficiency of Mylip gene in ECs exposed to targeting siRNA as compared to ECs exposed to scramble siRNA. ***p<0.001. C) Immunoblotting showing the impact of Mylip knockdown on basal expression of LDL-R on vascular ECs. | | |

|   |
| --- |
| **Supplementary figure 4.** IL-6 classic-signaling ECs does not alter LDL-C uptake. Reduction in LDL-R expression following IL-6 trans-signaling activation in ECs is blunted by co-stimulation with sgp130FC. Histogram and a bar graph showing LDL-C uptake using flowcytometry in ECs after 48h of treatment with IL-6 (100ng/ml) with or without sIL-6R (100ng/ml). **p<0.01. |

|  |  |
| --- | --- |
| **Supplementary figure 5.** Bar graphs showing gene expression of CD36 and CXCL16 in in vascular ECs treated with IL-6 (100ng/ml) in combination with sIL-6R (100ng/ml). **p<0.01. | |

|  |  |
| --- | --- |
| **Supplementary figure 6.** Bar graphs showing knockdown efficiency of CD36 and CXCL16 genes in ECs exposed to respective targeting siRNAs as compared to ECs exposed to scramble siRNA. ***p<0.001. | |

**Supplementary figure 7.** Representative histogram and bar graph showing Texas Red-Dextran (70kDa) uptake in ECs assessed using flowcytometry after 48h of treatment with IL-6+sIL-6R (both at 100ng/ml). Cells were incubated with EIPA (25µM) for the duration of the dextran uptake (1h).
